# Natural History of Glaucoma Progression in the DBA/2J Model: Early Contribution of Müller Cell Gliosis

**DOI:** 10.3390/cells12091272

**Published:** 2023-04-27

**Authors:** Rosario Amato, Maurizio Cammalleri, Alberto Melecchi, Paola Bagnoli, Vittorio Porciatti

**Affiliations:** 1 Department of Biology, University of Pisa, 56127 Pisa, Italy; maurizio.cammalleri@unipi.it (M.C.); a.melecchi@student.unisi.it (A.M.); paola.bagnoli@unipi.it (P.B.); 2 Bascom Palmer Eye Institute, University of Miami Miller School of Medicine, Miami, FL 33136, USA; vporciatti@med.miami.edu

**Keywords:** inherited model, intraocular pressure, vision loss, glial cell reactivity, retinal vasculature, blood–retinal barrier

## Abstract

Glaucoma is a chronic optic neuropathy characterized by progressive degeneration of retinal ganglion cells (RGCs). Elevated intraocular pressure (IOP) and the resulting mechanical stress are classically considered the main causes of RGC death. However, RGC degeneration and ensuing vision loss often occur independent of IOP, indicating a multifactorial nature of glaucoma, with the likely contribution of glial and vascular function. The aim of the present study was to provide a comprehensive evaluation of the time course of neuro–glial–vascular changes associated with glaucoma progression. We used DBA/2J mice in the age range of 2–15 months as a spontaneous model of glaucoma with progressive IOP elevation and RGC loss typical of human open-angle glaucoma. We found that the onset of RGC degeneration at 10 months of age coincided with that of IOP elevation and vascular changes such as decreased density, increased lacunarity and decreased tight-junction protein zonula occludens (ZO)-1, while hypoxia-inducible factor (HIF)-1α and vascular endothelial growth factor (VEGF) were already significantly upregulated at 6 months of age together with the onset of Müller cell gliosis. Astrocytes, however, underwent significant gliosis at 10 months. These results indicate that Müller cell activation occurs well before IOP elevation, with probable inflammatory consequences, and represents an early event in the glaucomatous process. Early upregulation of HIF-1α and VEGF is likely to contribute to blood retinal barrier failure, facilitating RGC loss. The different time courses of neuro–glial–vascular changes during glaucoma progression provide further insight into the nature of the disease and suggest potential targets for the development of efficient therapeutic intervention aside from IOP lowering.

## 1. Introduction

Glaucoma is one of the leading causes of vision loss in the world. Recent studies have shown that the pathogenesis of glaucoma is complex and it involves multiple factors that are often associated with intraocular pressure (IOP), although age is a major risk factor for glaucoma progression by contributing to the vulnerability of retinal ganglion cells (RGCs) to the damage of elevated IOP [1,2].

IOP elevation depends on an increased outflow resistance through the pressure-dependent trabecular outflow system, and it is the only modifiable risk factor. Current pharmacological intervention for glaucoma therapy is virtually limited to eye drops that reduce IOP [3]. However, the significant but not resolutive effects of hypotensive treatment as well as the existence of a significant percentage of IOP-independent forms of glaucoma highlight the limited knowledge of the physiopathological phenomena driving progressive retinal ganglion cell (RGC) death.

Animal models of glaucoma have facilitated major advances in our understanding of glaucoma, although each of them has its advantages and limitations. The DBA/2J (D2) inbred mouse strain is the most commonly used genetic model of glaucoma [4,5,6]. The D2 mouse develops anterior-segment abnormalities, including iris atrophy and pigment dispersion, leading to the occlusion of ocular drainage structures and subsequent IOP increase. In this strain, there is a gradual increase in IOP with age, associated with progressive loss of RGCs and optic nerve axons by 9 months of age [5,7,8]. Interestingly, RGC dysfunction non-invasively detected by pattern electroretinogram (PERG) has been demonstrated well before IOP elevation and RGC loss [9], thus mimicking the human condition in which the progressive dysfunction of RGCs precedes their structural loss by several years in suspected glaucoma [10]. Decreased PERG responses have been interpreted as dysfunction of RGCs and their axons possibly associated with failure of neurotrophic support [11,12] and age-related mitochondrial abnormalities [13].

Glial and vascular factors may also play an important role in RGC susceptibility to glaucomatous degeneration independent of IOP [14]. In fact, spontaneously occurring small fluctuations in IOP have been shown to generate chronic, low-grade ischemia–reperfusion injury, leading to blood vessel narrowing and reduced vessel density that progressively establish oxidative stress and trigger RGC loss [15]. Vasculature regression has been demonstrated in patients with diagnosed hypertensive glaucoma [16,17] and has been reported over a limited time window in glaucoma models soon after acute IOP elevation [18,19,20,21].

In addition to vessel regression, clinical and preclinical studies have demonstrated that glial cells undergo progressive gliosis [22], which contributes to RGC death through the release of pro-inflammatory cytokines independent of IOP increase [23], further supporting the role of neuroinflammation in RGC degeneration [24]. Gliosis may contribute to vascular dysregulation by upregulating hypoxia-inducible factor (HIF)-1 and its transcriptional targets, including vascular endothelial growth factor (VEGF), involved in the regulation of vessel density and permeability [25]. The HIF-1/VEGF axis is known to be upregulated in glaucoma models as a survival response to glaucomatous stress, although its persistent increase might induce alterations in blood–retinal barrier (BRB) integrity [26,27].

In the present study, the D2 model has been used to investigate whether the age-dependent RGC degeneration is temporally related to glial–vascular dysfunction. With this aim, we analyzed the age-dependent alterations in vasculature density in relation to astrocyte and Müller cell reactivity, along with the temporal profile of RGC loss and IOP elevation. Macroglial cell dysfunction was also correlated with progressive alterations in the levels of VEGF and its main transcriptional regulator HIF-1 in order to establish a link between glial cell damage and BRB dysfunction.

## 2. Materials and Methods

### 2.1. Animals

All procedures were performed in compliance with the ARVO Statement for the Use of Animals in Ophthalmic and Vision Research, EU Directive (2010/63/EU), and the Italian guidelines for animal care (DL 26/14; permission number: 349/2018-PR). A total of 60 D2 mice of both sexes (Charles River Laboratories, Calco, Italy) were used. They were kept in a regulated environment (23 ± 1 °C, 50 ± 5% humidity) with a 12 h light/dark cycle (lights on at 8:00 a.m.) and food and water ad libitum. Based on the D2 temporal profiling of age-dependent RGC dysfunction and IOP elevation [9], mice were divided into four different groups at 2, 6, 10 or 15 months of age in order to cover both the normotensive and the hypertensive stage of glaucoma (15 mice for each group). Two-month-old D2 mice were taken as controls in accordance with Saleh et al. [9].

### 2.2. Assessment of IOP

The mice of each age group underwent IOP recording via rebound tonometry. The mice were gently restrained on an adjustable stand and IOP was measured with a tonometer (TonoLab, Colonial Medical Supply, Franconia, NH, USA) measuring the rebound movement of a rod probe gently interacting with the eye. This movement was detected by a solenoid inside the instrument, and the motion parameters of the probe were monitored. The software was preprogrammed for 5 consecutive measurements per eye to obtain a reliable measure of IOP. After the assessment of IOP, mice from each age group were immediately euthanized by cervical dislocation and retinas were dissected for morphological and molecular analyses, while a separate group of mice underwent Evans blue dye perfusion and subsequent euthanasia for the analysis of vascular leakage.

### 2.3. Immunofluorescence

Retinas were isolated and immersion fixed for 2 h at 4 °C in 4% paraformaldehyde in 0.1 M phosphate-buffered saline (PBS) and then stored at 4 °C in 25% sucrose in 0.1 M PBS until used for immunofluorescence or isolectin B4 staining analysis (see Section 2.4). Six retinas for each group were used for RGC immunostaining using a guinea pig polyclonal anti-RNA-binding protein with multiple splicing (RBPMS, ABN1376, dilution 1:100; Merck, Darmstadt, Germany). An additional 6 retinas were used for astrocyte and Müller cell immunostaining using rabbit monoclonal anti-glial fibrillary acidic protein (GFAP, ab207165, dilution 1:400; Abcam, Cambridge, UK). Both RBPMS and GFAP antibodies were diluted in PBS containing 5% bovine serum albumin (BSA) and 2% Triton X-100. After overnight incubation at 4 °C, retinas were rinsed in PBS and incubated with FITC-conjugated anti-guinea pig secondary antibody (F6261; Merck, Darmstadt, Germany) or Alexa Fluor 555-conjugated anti-rabbit secondary antibody (A-32727; Molecular Probes, Eugene, OR, USA), respectively. They were both diluted 1:200 in PBS containing 5% BSA and 2% Triton X-100. Finally, retinas were rinsed in PBS and flat-mounted on polarized glass slides with the RGC layer facing up. Images from 4 radial areas (440 × 330 µm) in both the central and peripheral retina (center: 500 µm from the optic nerve head; periphery: 500 µm from the peripheral edge) were acquired using an epifluorescence microscope (Ni-E; Nikon-Europe, Amstelveen, The Netherlands) equipped with a digital camera (DS-Fi1c camera; Nikon-Europe, Amstelveen, The Netherlands). RGC density was measured as the average of RBPMS immunopositive somata per mm^2^. GFAP immunostaining was measured as the average of the mean gray levels normalized to the background using ImageJ software (U. S. National Institutes of Health, Bethesda, MD, USA; version 2.9.0). Z-stack digital images were collected at every 1 µm depth within 25 µm from the ganglion cell layer (GCL) focal plane for astrocytes or an additional 75 µm below for Müller cells. Convolution filtering and 3D reconstruction was performed using ImageJ software. Whole-mount images showing astrocytes and Müller cells in the same area were obtained by converting Z-stacks into 2D images using the “maximum intensity projection” function in ImageJ. Two-dimensional images were then merged after applying false colors to distinguish the GFAP immunoreactivity of astrocytes (red) from that of Müller cells (green).

### 2.4. Isolectin B4 Staining of Retinal Vessels

To visualize retinal vessels, 6 retinas for each age group were incubated with FITC-conjugated isolectin B4 (1:200; Vector Laboratories, Burlingame, CA, USA) diluted in PBS containing 5% BSA and 2% Triton X-100. After 48 h incubation at 4 °C, retinas were rinsed in PBS and flat-mounted on polarized glass slides with the RGC layer facing up. Images of retinal vessels were obtained following scanning acquisition with a 10x Plan-Apochromat objective using an epifluorescence microscope (Ni-E; Nikon-Europe, Amstelveen, The Netherlands) equipped with a digital camera (DS-Fi1c camera, Nikon-Europe) and subsequent automatic digital reconstruction with NIS-Elements software (Nikon-Europe, Amstelveen, The Netherlands). Angio Tool software was used to quantify vessel density as percent of the area occupied by vessels and lacunarity as the size distribution of gaps or lacunae between vessels [28]. Values of vessel density and lacunarity were normalized to those measured at 2 months.

### 2.5. Analysis of BRB Leakage Using Evans Blue Dye

For inner BRB assessment, 3 mice for each age group were anesthetized by intraperitoneal injection of avertin (1.2% tribromoethanol and 2.4% amylene hydrate in distilled water, 0.02 mL/g body weight; Sigma-Aldrich, St. Louis, MO, USA). Mice underwent systemic perfusion with 0.5% Evans blue dye (Sigma-Aldrich, St. Louis, MO, USA) in PBS intracardially injected into the left ventricle for 10 min. Then, mice were euthanized and explanted retinas were flat-mounted onto glass slides. Retinas were examined using an epifluorescence microscope (Ni-E; Nikon-Europe, Amstelveen, The Netherlands) equipped with a 10x Plan-Apochromat objective and a digital camera (DS-Fi1c camera, Nikon-Europe, Amstelveen, The Netherlands).

### 2.6. Western Blot 

Six retinas per age group were lysed with RIPA lysis buffer (Santa Cruz Biotechnology, Dallas, TX, USA) supplemented with phosphatase and proteinase inhibitor cocktails (Roche Applied Science, Indianapolis, IN, USA). Retina lysates were analyzed for protein concentration with a Micro BCA protein assay (Thermo Fisher Scientific, Waltham, MA, USA). Then, 40 µg of proteins per sample were separated using SDS-PAGE (4–20%; Bio-Rad Laboratories, Inc., Hercules, CA, USA) to be transblotted onto nitrocellulose membranes (Bio-Rad Laboratories, Inc., Hercules, CA, USA). Membranes were blocked with 5% skim milk for 1 h at room temperature and then incubated overnight at 4 °C with the same blocking solution containing rabbit monoclonal antibody anti-vascular endothelial growth factor (VEGF, ab214424, dilution 1:100, Abcam, Cambridge, UK), anti-hypoxia-inducible factor-1α (HIF-1α, ab179483, dilution 1:1000, Abcam, Cambridge, UK) and anti-zonula occludens-1 (ZO-1, 40-2200, dilution 1:500, Thermo Fisher Scientific, Waltham, MA, USA), or mouse monoclonal antibody anti-β actin (A2228, dilution, Sigma-Aldrich). Thereafter, membranes were incubated for 2 h at room temperature with appropriate HRP-conjugated secondary anti-mouse or anti-rabbit (1:5.000) antibodies. Blots were developed using Clarity Western enhanced chemiluminescence substrate (Bio-Rad Laboratories, Inc.) and the images were acquired using ChemiDoc XRS+ (Bio-Rad Laboratories, Inc., Hercules, CA, USA). The optical density (OD) relative to the target bands (Image Lab 3.0 software; Bio-Rad Laboratories, Inc., Hercules, CA, USA) was normalized to the corresponding OD of β-actin as loading control.

### 2.7. Statistical Analysis

Data were analyzed with the Shapiro–Wilk test to verify their normal distribution. Differences among groups were analyzed with one-way ANOVA followed by Tukey post-hoc test or two-way ANOVA followed by Bonferroni’s multiple comparison post-test using Prism 8.0.2 (GraphPad Software, Inc., San Diego, CA, USA). Data are expressed as box-and-whisker plots, with boxes representing the 25–75th percentiles and whiskers indicating their minimum and maximum value. Differences with *p* < 0.05 were considered significant. To correlate the temporal profile of IOP-associated RGC loss with that of glial-vascular dysfunction, spline regression curves were created using JMP Pro 14.2 (SAS Institute Inc., Cary, NC, USA). Variables are expressed as percentage of the relative dynamic range (max–min). Shaded areas represent the 95% confidence interval of the spline curves.

## 3. Results

### 3.1. Age-Dependent Profiling of IOP Increase, RGC Loss and Vascular Regression

**Figure 1:**
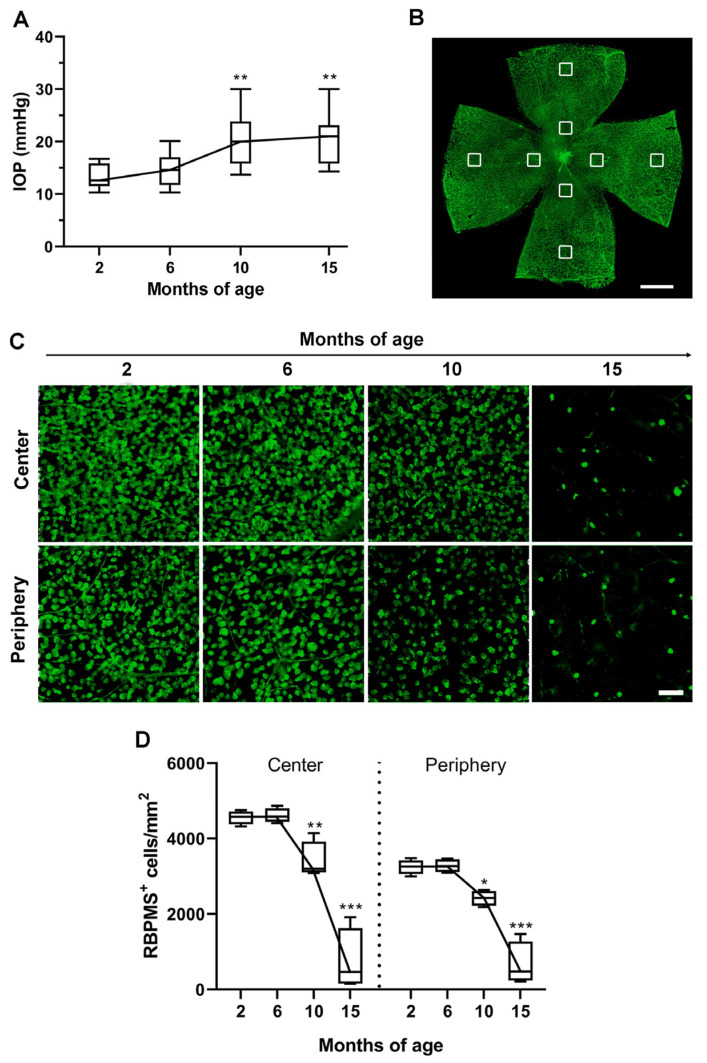
Time course of intraocular pressure (IOP) elevation in respect to retinal ganglion cell (RGC) loss. (**A**) Longitudinal evaluation of IOP levels at 2, 6, 10 and 15 months of age as assessed by rebound tonometry. (**B**) RNA-binding protein with multiple splicing (RBPMS) immunolabeled whole mount showing the localization of the sampling areas (at 500 µm from the optic nerve head for the center or 500 µm from the peripheral edge for the periphery) in each of the four retinal quadrants. Scale bar: 1 mm. (**C**) Representative high-magnification images of RBPMS immunostaining from the central or peripheral retina at 2, 6, 10 and 15 months of age. Scale bar: 50 µm. (**D**) Quantitative analysis of RBPMS^+^ cell density. Data derive from the average of four areas sampled in the retinal center or periphery. Box plots represent the statistical distribution of six independent samples. Differences among groups were tested using one-way ANOVA followed by Tukey’s multiple comparison post-hoc test. * *p* < 0.05, ** *p* < 0.01, *** *p* < 0.001 vs. 2 months of age.

The relative occurrence of RGC degeneration and vascular alterations over the progressive IOP increase was evaluated by profiling IOP levels, RGC density and vessel density in D2 retinas at 2, 6, 10 and 15 months of age. Age-dependent alterations in IOP levels typical of the D2 model were profiled by plotting IOP recordings obtained from each age group (Figure 1A). In accordance with Saleh et al. [9], IOP was maintained at baseline levels over the period from 2 to 6 months. Baseline IOP levels recorded at 2 months (13.38 ± 0.76 mmHg) were comparable to those recorded at 6 months (14.56 ± 0.86 mmHg, *p* = 0.91 vs. 2 months). At 10 months, IOP (19.94 ± 1.38 mmHg) increased by about 49% as compared to the baseline level at 2 months (*p* = 0.0098 vs. 2 months) to be maintained up to 15 months when IOP levels (20.5 ± 1.66 mmHg) were still higher than the baseline (*p* = 0.0031 vs. 2 months) and comparable to those at 10 months (*p* = 0.98 vs. 10 months). As shown in Figure 1B, RGC density over time was assessed in the central and peripheral retina using RBPMS immunostaining. In line with previous results [8,29,30], RGC density did not differ between 2 and 6 months of age, while RGC loss was evident at 10 months in both the central and peripheral retina. At 15 months, only sparse RGCs with shrunken cell bodies could be seen (Figure 1C). As shown in Figure 1D, quantitative analysis of RGC density revealed that the baseline values at 2 months in both the central (4556.32 ± 89.06 cells/mm^2^) and peripheral retina (3246.64 ± 99.03 cells/mm^2^) were comparable to those at 6 months (center: 4609.76 ± 96.19 cells/mm^2^; periphery: 3273.55 ± 88.23 cells/mm^2^). RGC loss was clearly evident at 10 months, when the cell density in the central (3405.95 ± 248.31 cells/mm^2^) and peripheral (2414.81 ± 102.05 cells/mm^2^) retina proportionally decreased by about 25% as compared with that at 2 months (center: *p* = 0.0041; periphery: *p* = 0.041 vs. 2 months). RGC density further decreased at 15 months (center: 749.15 ± 412.17 cells/mm^2^; periphery: 660.57 ± 286.52 cells/mm^2^), reaching about 80% loss as compared to 2 months.

**Figure 2:**
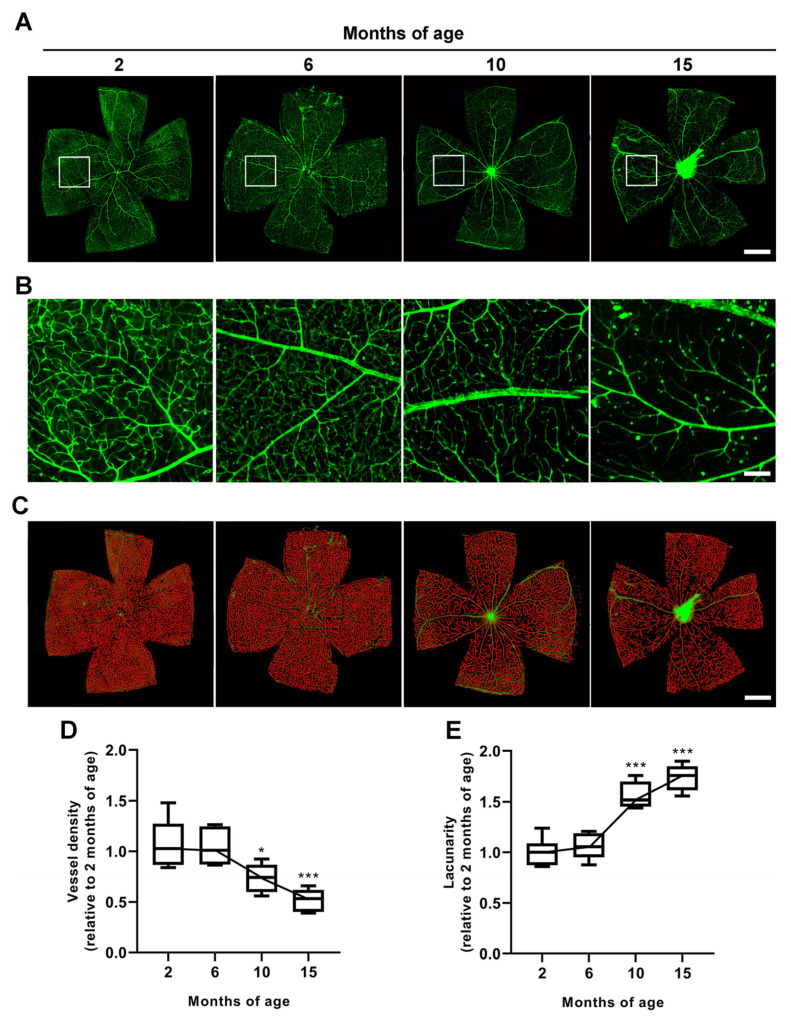
Age-dependent alterations in retinal vessel density and lacunarity as assessed by isolectin B4 staining in whole-mount retinas. (**A**) Representative images showing the superficial vascular plexus stained with isolectin B4 at different ages. Scale bar: 1 mm. (**B**) Corresponding high-magnification images of superficial vessels in the mid-peripheral retina (1 mm from the optic nerve head). Scale bar: 150 μm. (**C**) Images of the whole mounts shown in A depicting the skeleton of the vasculature (red) in which vessel density was quantified as percent of the area occupied by vessels and lacunarity as the size distribution of gaps or lacunae between vessels. (**D**,**E**) Quantitative analysis of time-dependent variation in vessel density and lacunarity. Box plots represent the statistical distribution of six independent samples. Differences among groups were tested using one-way ANOVA followed by Tukey’s multiple comparison post-hoc test. * *p* < 0.05, *** *p* < 0.01 vs. 2 months of age.

Assessment of vessel density in the superficial vascular plexus revealed a progressive reduction temporally related to IOP increase. Representative images of retinal whole mounts stained with isolectin B4 are shown in Figure 2A. Vessel density did not change from 2 to 6 months of age, while vascular regression was evident from 10 months, as shown by high magnification in Figure 2B. Vessel density was also evaluated by the parametrical analysis of vessel lacunarity, which is indicative of the gap size between the vessels, thus allowing the evaluation of microvascular geometry in the retina [28]. The retinal whole mounts of Figure 2C show the skeleton of the vasculature at different ages at which vessel density was quantified as percent of the area occupied by vessels and lacunarity as the size distribution of gaps or lacunae between vessels. In Figure 2D, the parametrical analysis of the area covered by retinal vessels demonstrated a comparable vascular density between 2 and 6 months (*p* = 0.98 vs. 2 months). At 10 months, vessel density decreased by 0.73 ± 0.056-fold as compared to baseline levels at 2 months (*p* = 0.015 vs. 2 months), and then progressively decreased up to 15 months, when the area covered by vessels was 0.52 ± 0.045-fold lower than that at 2 months (*p* = 0.00014 vs. 2 months). As shown in Figure 2E, vessel lacunarity at 6 months was comparable to that at 2 months (*p* = 0.87 vs. 2 months) and then progressively increased from 10 to 15 months by 1.56 ± 0.053- and 1.74 ± 0.051-fold higher, respectively, than the baseline values at 2 months (*p* < 0.0001 vs. 2 months).

### 3.2. Age-Dependent Profiling of Astrocyte and Müller Cell Reactivity

Both astrocytes and Müller cells provide homeostatic and metabolic support to the retina required for neuronal activity, with astrocytes in close contact with blood vessels and Müller cells providing radial support. That gliosis contributes to RGC loss over glaucoma progression is well established [31], although the age-dependent contribution of astrocytes and Müller cells remains to be fully clarified.

**Figure 3:**
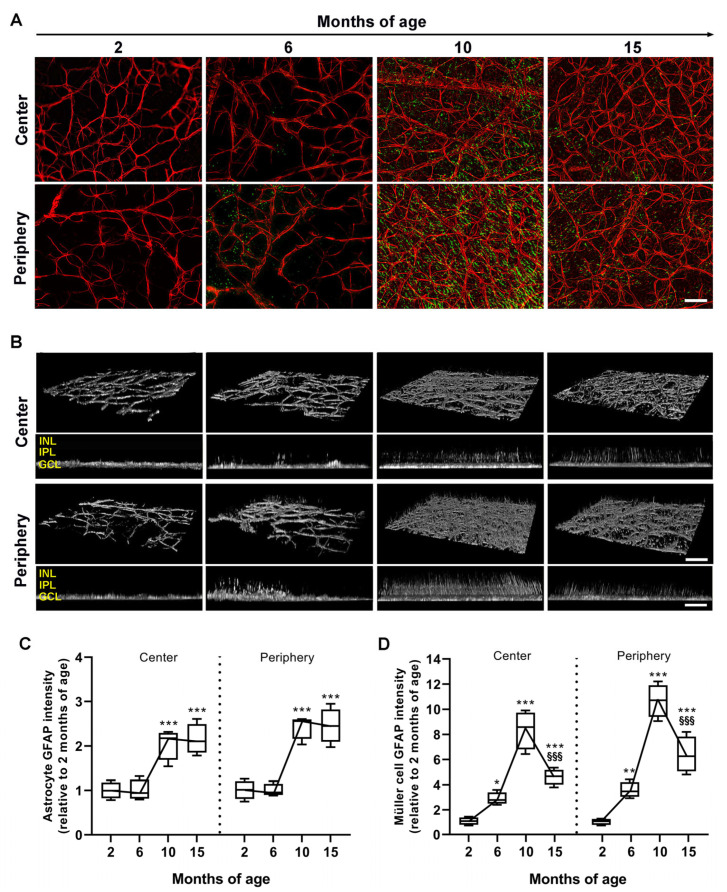
Age-dependent variations in astrocyte and Müller cell reactivity. (**A**) Representative images of retinal whole mounts showing glial fibrillary acidic protein (GFAP) immunolabeling in astrocytes (red) and Müller cells (green) in the central and peripheral retina. Scale bars: 50 µm. (**B**) Corresponding 3D reconstructions displaying reactive astrocyte and Müller cell processes, which are better seen by Z-stack projections. Scale bars: 50 µm (3D reconstructions), 70 µm (Z-stack projections). (**C**,**D**) Quantitative analysis of astrocyte and Müller cell GFAP fluorescence intensity in the center and the periphery. Data derive from the average of four areas sampled in the retinal center or periphery. Box plots represent the statistical distribution of six independent samples. Differences among groups were tested using two-way ANOVA followed by Bonferroni multiple comparison post-hoc test. * *p* < 0.05, ** *p* < 0.01, *** *p* < 0.001 vs. 2 months of age; ^§§§^ *p* < 0.001 vs. 10 months of age. INL, inner nuclear layer; IPL, inner plexiform layer; GCL, ganglion cell layer.

In whole-mount retinas, the time course of astrocyte (in red) and Müller cell (in green) gliosis was profiled by analyzing the immunostaining pattern of GFAP, an intermediate filament protein that is a typical marker of glial cell reactivity (Figure 3A). Under normal conditions, GFAP is constitutively expressed by astrocytes, while non-reactive Müller cells are generally not visualized by GFAP immunostaining [32]. GFAP immunoreactivity at 2 months was limited to astrocytes, which are located in the GCL and display normal morphology with well-organized branching radiating from the cell body. At 6 months, astrocytes were almost unaltered in their morphology, while at 10 months, GFAP-immunopositive astrocytes were characterized by hypertrophic and poorly organized branching typical of their reactive phenotype, and maintained this morphology through 15 months. As shown by 3D reconstructions and Z-projections of the inner retina (Figure 3B), at 2 months no GFAP immunoreactivity could be attributed to Müller cells, as demonstrated by the negligible presence of GFAP orthogonal processes. At 6 months, GFAP-positive orthogonal profiles indicative of reactive Müller cells could be observed to further increase in density at 10 months, mainly in the peripheral retina. In contrast, at 15 months, GFAP orthogonal processes were decreased but still detectable. Immunofluorescence quantification relative to astrocytes is shown in Figure 3C. At 6 months, the intensity of GFAP immunoreactivity did not differ from that measured at 2 months (center: *p* = 0.98; periphery: *p* = 0.97 vs. 2 months of age). Compared with 2 months, at 10 months, GFAP immunoreactivity significantly increased in both the central (2.05 ± 0.18-fold, *p* = 0.0001 vs. 2 months) and peripheral retina (2.44 ± 0.14-fold, *p* < 0.0001 vs. 2 months) to become similar to that measured at 15 months (center: 2.15 ± 0.17-fold, *p* < 0.0001; periphery: 2.45 ± 0.20-fold, *p* < 0.0001 vs. 2 months). Immunofluorescence quantification relative to Müller cells is shown in Figure 3D. GFAP intensity was found to increase as early as 6 months as compared with 2 months in both the central (2.89 ± 0.25-fold, *p* = 0.039 vs. 2 months) and peripheral retina (3.57 ± 0.31-fold, *p* = 0.0025 vs. 2 months), but with significantly higher intensity in the periphery in respect to the center, which was maintained up to 15 months. GFAP intensity further increased up to 10 months, reaching a peak of 8.63 ± 0.57-fold in the center and 9.67 ± 0.72-fold in the periphery (vs. 2 months). At 15 months, GFAP intensity drastically decreased (center: 4.61 ± 0.32-fold, *p* < 0.0001; periphery: 6.38 ± 0.72-fold, *p* = 0.00013 vs. 10 months), but was still significantly higher than that at 2 months (center: *p* < 0.0001; periphery: *p* < 0.0001 vs. 2 months).

### 3.3. Age-Dependent Changes in VEGF Levels and Inner BRB Integrity

**Figure 4:**
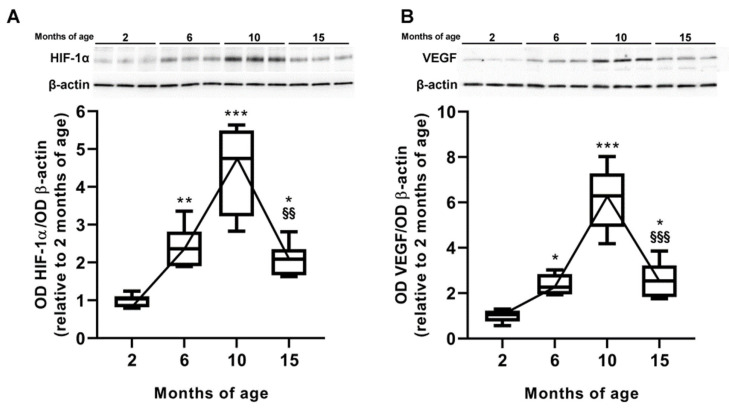
Age-dependent variations of hypoxia-inducible factor- (HIF)-1α and vascular endo-166 thelial growth factor (VEGF) levels. (**A**,**B**) Representative Western blots and densitometric analysis of HIF-1α and VEGF protein levels at different ages. β-actin was used as loading control. Box plots represent the statistical distribution of six independent samples. Differences among groups were tested using one-way ANOVA followed by Tukey’s multiple comparison post-hoc test. * *p* < 0.05, ** *p* < 0.01, *** *p* < 0.001 vs. 2 months of age; ^§§^ *p* < 0.01, ^§§§^ *p* < 0.001 vs. 10 months of age.

We verified whether HIF-1α and VEGF levels were related to glaucoma progression over time. As shown in Figure 4A, HIF-1α levels increased by 2.25 ± 0.28-fold from 2 to 6 months (*p* = 0.044 vs. 2 months), with a further increment at 10 months, reaching a peak of 4.46 ± 0.46-fold increase relative to 2 months (*p* < 0.0001 vs. 2 months). At 15 months, the levels of HIF-1α were lower than the peak levels at 10 months (*p* = 0.0014 vs. 10 months), but still 2.53 ± 0.29-fold higher than those at 2 months (*p* = 0.011 vs. 2 months). As shown in Figure 4B, retinal levels of VEGF increased by 2.36 ± 0.19-fold as early as 6 months as compared to 2 months (*p* = 0.048 vs. 2 months), and then progressively increased up to 10 months (6.16 ± 0.55-fold higher than those at 2 months; *p* < 0.0001). At 15 months, VEGF levels were lower than those at 10 months (*p* < 0.0001 vs. 10 months of age), but still 2.59 ± 0.34-fold higher than those at 2 months (*p* = 0.018 vs. 2 months).

**Figure 5:**
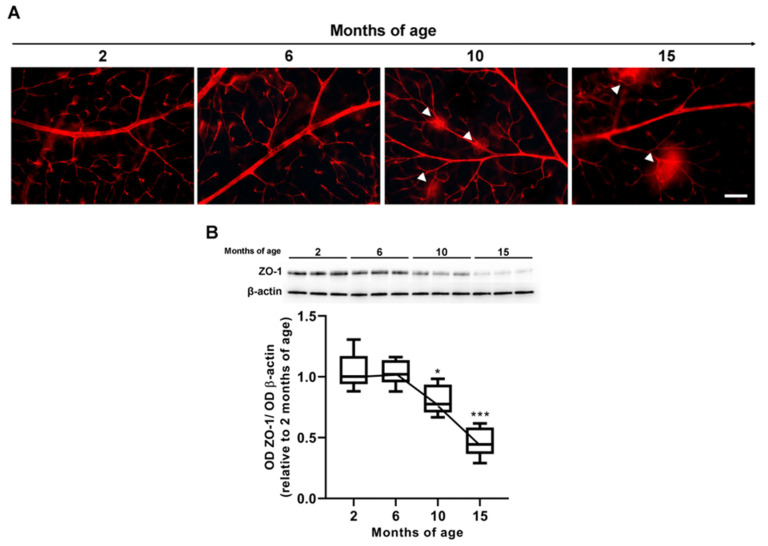
Analysis of inner blood retinal barrier (BRB) integrity at increasing ages. (**A**) Representative images displaying retinal vessels perfused with Evans blue dye at different ages. Arrowheads indicate dye extravasation as a consequence of plasma protein leakage. Scale bar: 50 µm. (**B**) Representative Western blots and densitometric analysis of the tight-junction protein zonula occludens (ZO)-1. β-actin was used as loading control. Box plots represent the statistical distribution of independent samples. Differences among groups were tested using one-way ANOVA followed by Tukey’s multiple comparison post-hoc test. * *p* < 0.05, *** *p* < 0.01 vs. 2 months of age.

Associated with VEGF increase, inner BRB underwent progressive dysfunction, characterized by the occurrence of vascular leakage as demonstrated by qualitative assessment of Evans blue dye extravasation (Figure 5A). In particular, the dye was retained in the vascular lumen for up to 6 months, while its extravasation could be observed at 10 months and then increased up to 15 months. As also shown here, vascular leakage was temporally correlated with levels of the tight-junction protein ZO-1 (Figure 5B). Its levels at 6 months did not differ from those at 2 months (*p* = 0.96 vs. 2 months), while ZO-1 levels at 10 months were 0.81 ± 0.049-fold lower than those at 2 months (*p* = 0.017 vs. 2 months). A further 0.45 ± 0.049-fold decrease could be observed at 15 months (*p* < 0.0001 vs. 2 months).

### 3.4. Comparison between Time Courses of RGC, IOP and Retinal Factors

**Figure 6:**
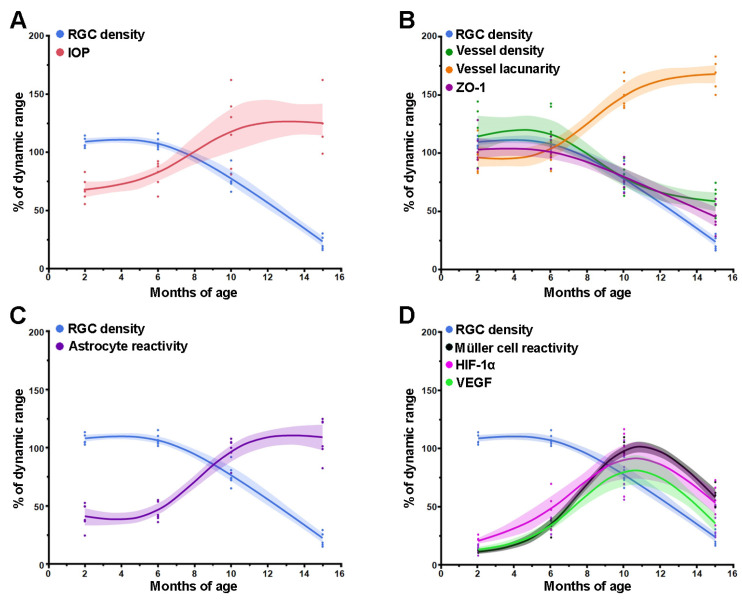
Spline regression curves comparing the temporal profile of RGC density with major events characterizing glaucoma progression in D2 retinas. (**A**) RGC density in respect to IOP elevation; (**B**) RGC density in respect to vessel density/lacunarity and BRB leakage as determined by densitometric analysis of ZO-1 protein levels; (**C**) RGC density in respect to astrocyte reactivity; (**D**) RGC density in respect to Müller cell gliosis and its associated HIF-1α/VEGF accumulation. Data are expressed as percentage of their dynamic range. The shaded areas represent the 95% confidence interval of spline regression curves.

Comparison of the temporal profiles of multiple factors potentially contributing to RGC death in glaucoma may provide mechanistic insight into RGC death and help identify early changes in factors that may represent a treatable target to prevent the disease. As different measures have different scales, data have been normalized by dividing experimental data for each measure by their corresponding dynamic range (maximum value minus minimum value). In Figure 6, the time course of RGC loss has been taken as reference and compared with those of other measures. As shown in Figure 6A, the time-course of RGC loss is inversely associated with the temporal profile of IOP. In Figure 6B, the time-course of RGC loss overlaps that of vessel density and leakage, while that of vessel lacunarity is mirror-symmetric. The profile of astrocyte reactivity (Figure 6C), also appears similar to that of IOP (Figure 6A) and vessel lacunarity (Figure 6B). As all these variables have virtually comparable time courses (although of different direction), it is impossible to draw causal conclusions about mutual relationships. A cluster of factors (Müller reactivity, VEGF, HIF-1α), however, have a temporal profile that markedly differs from that of RGCs (Figure 6D), with earlier onset and increase to a peak and a late decline that overlaps that of RGC death. These factors precede RGC death and are possibly causative factors.

## 4. Discussion

In the present study, IOP and neuro–glial–vascular factors have been temporally profiled in D2 glaucoma from 2 to 15 months in order to provide further insight into processes that may possibly be targeted by preventive or protective approaches. As shown by the present results, the onset of IOP elevation and RGC loss occurs after 6 months of age, in keeping with a large body of literature [8,29,30]. IOP elevation may contribute to RGC death via mechanical stress or exacerbate dysregulatory factors, reducing ocular blood flow [33]. In parallel with IOP elevation, retinal vessel density decreases in concomitance with lacunarity increase, which reflects a non-uniform vessel organization. This is in keeping with previous findings in preclinical models demonstrating reduced density of retinal capillaries at the level of the optic nerve head as a result of acute IOP increase [21].

In addition to vascular dysfunction, glial cell reactivity may contribute to RGC death by affecting the relationship between vessels and neurons [22,31,34]. Of the macroglial cells, both astrocytes and Müller cells are important contributors to the pathophysiological processes leading to progressive glaucomatous RGC loss [34]. As demonstrated by the present results, astrocytes become reactive in concomitance with IOP increase and RGC loss, indicating their possible role in exacerbating RGC degeneration. In effect, reactive astrocytes have been shown to amplify neuroinflammatory response by releasing pro-inflammatory cytokines [35], which contributes to RGC axon degeneration and structural remodeling of the optic nerve under sustained IOP elevation [36]. In contrast, Müller cell gliosis precedes IOP increase, in line with previous findings from D2 mice [37]. In particular, Müller cell gliosis in the retinal periphery is higher than in the center, in line with the finding that peripheral RGCs are more susceptible to death than in the center, with RGC loss beginning in the peripapillary retina and then spreading peripherally [7,38]. As a rapid response to retinal injury, early stages of gliosis have neuroprotective potential to halt disease progression by buffering extra K^+^ levels, regulating glutamate uptake, and releasing a variety of factors that protect neurons from degeneration [39]. On the other hand, persistent Müller cell gliosis can be detrimental for RGC function and survival by triggering sustained inflammation and RGC degeneration. In particular, reactive Müller cells have been shown to reduce the degradation of glutamic acid, thus inducing excitotoxic damage of RGCs [40]. In addition, Müller cell gliosis can further contribute to RGC damage by enhancing neuroinflammation through the release of pro-inflammatory cytokines [41], which may act as main astrocyte activators [31]. Furthermore, Müller cell reactivity induces alterations in K^+^ siphoning, thus leading to cation dyshomeostasis [42]. As K^+^ siphoning has been shown to contribute to PERG generation [43,44], early Müller cell reactivity is likely to contribute to the progressive PERG decrease that has been found to precede IOP elevation [9].

An additional sign of glaucoma progression is the accumulation of both HIF-1α and VEGF, to which IOP and non-IOP related mechanisms are likely to contribute. In fact, IOP elevation has been found, by dysregulating blood flow, to cause a relative hypoxia that contributes to HIF-1α activation and its downstream upregulation of VEGF by Müller cells and astrocytes [45]. On the other hand, early reactive gliosis is critically involved in the activation of the HIF-1α/VEGF pathway independent of IOP elevation [34]. Although HIF-1α increase occurs well before VEGF upregulation, the fact that both markers vary in concert during the progression of glaucoma can be due to the time scale of the present analysis, which overcomes the temporal sequence of VEGF accumulation. Indeed, VEGF gene transcription by HIF-1α takes a few hours after hypoxic injury to the retina [46,47].

Through the production of VEGF, glial cells act at multiple levels, with a major regulatory role in vascular permeability [48]. In fact, increased Müller cell-derived VEGF results in depletion of tight-junction proteins in endothelial cells such as occludin and ZO-1, thus impairing BRB function [49]. In this line, dysregulated neurovascular integrity is a major factor driving RGC death [50], although glaucoma-associated neuroinflammation contributes to RGC damage and limits visual recovery [26].

Aside from affecting vessel permeability, VEGF accumulation may serve as a protective mechanism to compensate for RGC dysfunction. In glaucoma models, for instance, VEGF acts as a neuroprotective factor to promote RGC survival [51]. A protective role of VEGF in glaucoma patients is supported by the finding that elevated VEGF is present in the aqueous humor, thus suggesting its potential neuroprotective role [52]. As shown by the present results, VEGF accumulation precedes RGC loss but coincides with dysfunctional RGC activity, indicating that VEGF overexpression and release are among the earliest responses to suffering retinal neurons. In this phase, VEGF upregulation would presumably serve as a protective strategy to counteract retinal cell damage. At increasing ages, VEGF increase may instead impair vessel permeability and BRB integrity.

## 5. Conclusions

The time course of RGC loss appears to be similar to the temporal profile of IOP increase and the time course of vessel regression/increased lacunarity and leakage as well as astrocyte reactivity, but not with the Müller cell gliosis and HIF-1α/VEGF-associated increase that precede IOP elevation and RGC death. This implies that abnormalities coinciding in time with RGC death may possibly represent exacerbating factors over the manifest illness stage, while events preceding RGC loss presumably contribute to the early phases of disease progression and are likely to represent specific stressors to be targeted by preventive and protective approaches. The present findings highlight the possibility that in addition to IOP lowering, the control of additional risk factors might be essential in mitigating the adverse effects of IOP increase on RGC health. Reactive Müller cell gliosis as an early support of RGC survival followed by accelerated degenerative processes is a common characteristic of retinal diseases, including diabetic retinopathy, in which Müller cell-mediated inflammation has been shown to play a prominent role in retinal cell loss [53]. In this respect, much effort is aimed at developing non-IOP-related neuroprotective interventions that can prevent neuronal cell loss in the retina. In particular, targeting glial cells may represent a new therapeutic strategy for treating glaucoma with Müller cells, which is expected to play a prominent role in the development of future therapeutic approaches to protecting RGCs in glaucoma [54]. In particular, selective Müller cell-specific targeting has the advantage of addressing a major source of neurotrophic factors through the latest phases of the disease [55]. In addition, the therapeutic potential of Müller glia appears attractive given their well-recognized neurotrophic role and potential stem/progenitor cell phenotype. In fact, Müller cells have been reported to display stem-cell characteristics that posit them as an interesting target for regenerative therapies aimed at stimulating intrinsic replacement of injured retinal neurons [56].

## Data Availability

The data presented in this study are available upon request from the corresponding author.
